# miR160 and miR166/165 Contribute to the *LEC2*-Mediated Auxin Response Involved in the Somatic Embryogenesis Induction in Arabidopsis

**DOI:** 10.3389/fpls.2017.02024

**Published:** 2017-12-11

**Authors:** Anna M. Wójcik, Michael D. Nodine, Małgorzata D. Gaj

**Affiliations:** ^1^Department of Genetics, University of Silesia, Faculty of Biology and Environmental Protection, Katowice, Poland; ^2^Gregor Mendel Institute, Austrian Academy of Sciences, Vienna Biocenter (VBC), Vienna, Austria

**Keywords:** AUXIN RESPONSE FACTOR10, ARF16, ARF17, LEAFY COTYLEDON2, miRNA, PHABULOSA, PHAVOLUTA, somatic embryogenesis

## Abstract

MicroRNAs are non-coding small RNA molecules that are involved in the post-transcriptional regulation of the genes that control various developmental processes in plants, including zygotic embryogenesis (ZE). miRNAs are also believed to regulate somatic embryogenesis (SE), a counterpart of the ZE that is induced *in vitro* in plant somatic cells. However, the roles of specific miRNAs in the regulation of the genes involved in SE, in particular those encoding transcription factors (TFs) with an essential function during SE including *LEAFY COTYLEDON2 (LEC2*), remain mostly unknown. The aim of the study was to reveal the function of miR165/166 and miR160 in the *LEC2*-controlled pathway of SE that is induced in *in vitro* cultured Arabidopsis explants.In ZE, miR165/166 controls the *PHABULOSA*/*PHAVOLUTA* (*PHB/PHV*) genes, which are the positive regulators of *LEC2*, while miR160 targets the *AUXIN RESPONSE FACTORS* (*ARF10, ARF16, ARF17*) that control the auxin signaling pathway, which plays key role in LEC2-mediated SE. We found that a deregulated expression/function of miR165/166 and miR160 resulted in a significant accumulation of auxin in the cultured explants and the spontaneous formation of somatic embryos. Our results show that miR165/166 might contribute to SE induction via targeting *PHB*, a positive regulator of LEC2 that controls embryogenic induction via activation of auxin biosynthesis pathway (Wójcikowska et al., 2013). Similar to miR165/166, miR160 was indicated to control SE induction through auxin-related pathways and the negative impact of miR160 on *ARF10/ARF16/ARF17* was shown in an embryogenic culture. Altogether, the results suggest that the miR165/166- and miR160-node contribute to the LEC2-mediated auxin-related pathway of embryogenic transition that is induced in the somatic cells of Arabidopsis. A model summarizing the suggested regulatory interactions between the miR165/166-PHB and miR160-ARF10/ARF16/ARF17 nodes that control SE induction in Arabidopsis was proposed.

## Introduction

The unique developmental plasticity of plant cells has been widely documented by the successful formulation of *in vitro* culture protocols that enable the efficient clonal propagation of numerous plant species (reviewed Misra and Saema, 2016). In a vast number of these protocols, plants are regenerated via somatic embryogenesis (SE), a unique developmental process in which already differentiated somatic cells undergo embryogenic transitions giving rise to somatic embryo production (reviewed in Altamura et al., 2016). Thus, the identification of the genetic networks that govern SE induction greatly contributes to both the understanding of the molecular mechanisms that control plant totipotency and the improvement of the plant micropropagation protocols. In numerous plants including *Arabidopsis thaliana* (Arabidopsis), zygotic embryos at precisely defined developmental stages provide the most efficient tissue to induce SE (Elithi and Stasolla, 2011; Wójcikowska and Gaj, 2016). In Arabidopsis, the culture of zygotic embryos at a late cotyledonary stage of development on an auxin medium has been recommended to induce SE for molecular studies on plant cell totipotency (Gaj, 2004). Using this model SE system has resulted in the remarkable progress in deciphering the genetic mechanisms that govern SE induction that has been achieved in recent years (reviewed in Wójcikowska and Gaj, 2016).

A predominant number of genes with a documented decisive role in SE induction encode transcription factors (TFs) including, BABY BOOM (BBM) (Boutilier et al., 2002), WUSCHEL (WUS) (Zuo et al., 2002), AGAMOUS-LIKE15 (AGL15) (Harding et al., 2003) and LEAFY COTYLEDON (LEC1, LEC2) (Stone et al., 2001; Gaj et al., 2005) (reviewed in Nowak and Gaj, 2016). Global analysis of SE-transcriptome in Arabidopsis indicated that in concert with extensive modulation of *TF* genes (Gliwicka et al., 2013) numerous miRNAs are differentially expressed in embryogenic culture (Szyrajew et al., 2017). Similar to Arabidopsis, differential expression of miRNAs was reported in the embryogenic cultures of other plants, including *Oryza sativa* (Chen et al., 2011), hybrid yellow poplar (Li et al., 2012), *Larix laptolerix* (Zhang et al., 2012), *Dimocarpus longan* (Lin and Lai, 2013), *Gossypium hirsutum* (Yang et al., 2013) and *Zea mays* (Chávez-Hernández et al., 2015). Thus, it is believed that in control of SE, like in other plant developmental processes including zygotic embryogenesis (ZE) (Jones-Rhoades et al., 2006) miRNAs are involved but the genes targeted by specific miRNA and their function in the mechanism governing embryogenic transition is mostly unknown.

Hence, identifying the miRNAs that regulate SE, extend our knowledge about the regulatory pathways controlling embryogenic transitions in somatic plant cells. The *LEC2* gene encodes a plant specific B3-domain TF (Harada, 2001) that is essential for SE induction. The expression level of *LEC2* was reported to positively impact embryogenic transition in somatic cells of Arabidopsis *in planta* and *in vitro* (Stone et al., 2001; Ledwon and Gaj, 2009) and transcripts of *LEC2* were found to accumulate in the explant cells undergoing SE induction in response to auxin treatment (Kurczynska et al., 2007; Ledwon and Gaj, 2011). The LEC2-mediated mechanism controlling SE induction involves the activation of the *YUCCA* genes that contribute to the auxin biosynthesis via tryptophan-dependent pathway (Wójcikowska et al., 2013). The upstream elements that regulate *LEC2* during SE induction remain unknown and among the candidates are PHABULOSA/PHAVOLUTA (PHB/PHV) proteins of the class III HOMEODOMAIN LEUCINE ZIPPER (HD-ZIP III) TF family that directly activate *LEC2* in vegetative development of Arabidopsis (Tang et al., 2012). Transcripts of *HD-ZIP III* genes are targeted by miR165/166 (Zhong and Ye, 2007). In Arabidopsis, two copies of the *MIR165* and six of the *MIR166* genes produce the mature miR165 and miR166 molecules that comprise the sequence of 21 nucleotides differing by one nucleotide (Reinhart et al., 2002). A role of miR165/166 in the regulation of *PHB* and *PHV* genes was revealed by biochemical and genetic analysis (Tang et al., 2003; Jung and Park, 2007) and the engagement of miR165/166-PHB/PHV in control of diverse developmental processes was indicated including radial pattering in shoots (McConnell et al., 2001), development of ovules (Sieber et al., 2004) and leaves (Mallory et al., 2004), xylem specification and differentiation of pericycle (Miyashima et al., 2011) and vascular tissues (Du and Wang, 2015). During ZE, *PHB*, and *PHV* are repressed by miR165/166 to properly regulate early patterning of embryos and prevent precocious expression of differentiation-promoting TFs (Grigg et al., 2009; reviewed in Jia et al., 2014). The embryos of gain-of function *phb-1d* mutant carrying defective miR165/166-binding site in the *PHB* gene have larger shoot meristem and radialized cotyledons (McConnell and Barton, 1998). Consistent with a phenotype of the *phb-1d* mutant embryos, the miR165/166-*PHB/PHV* module was shown to contribute to the establishment of bilateral symmetry and the shoot apical meristem (SAM) during ZE (Prigge et al., 2005; Smith and Long, 2010).

The miR160-mediated repression of *AUXIN RESPONSE FACTOR* TFs (ARFs), including *ARF10, ARF16*, and *ARF17* regulates several aspects of plant development. For example, miR160-directed repression of *ARF10* and *ARF16* regulates root cap, RAM and primary as well as lateral root development (Wang et al., 2005; Bustos-Sanmamed et al., 2013), floral organs and ovary patterning (Damodharan et al., 2016), and seed germination (Liu et al., 2007). Moreover, plants expressing miR160-resistant versions of *ARF17* had altered expression of the early auxin responsive genes and defective embryo, root, vegetative and floral organ development (Mallory et al., 2005). In addition, miR160/ARF17 controls pollen wall formation (Yang et al., 2013), and male sterility (Shi et al., 2015).

Floral organs in carpels (*foc*) mutants in the 3′ region of the *MIR160a* gene have reduced miR160 levels and exhibit abnormal cell divisions in the root meristem precursors and suspensor of ZEs (Liu et al., 2010). The expression of *ARF10, ARF16* and *ARF17* was distinctly affected in the *foc* mutant suggesting that miR160 controls zygotic embryo development through auxin signaling (Liu et al., 2010).

The differential expression of the *MIR160* and *MIRNA165/166* genes and their mature miR160 and miR166 products in the embryogenic culture of Arabidopsis (Szyrajew et al., 2017) motivated us to explore the function of miR165/166 and miR160 during SE induction. To this end, the candidate targets of miR165/miR166 (*PHB/PHV*) and miR160 (*ARF10/ARF16/ARF17*) were evaluated in SE culture of Arabidopsis with a disturbed expression/function of the studied genes. The relation between the expression level of miR165/166 and miR160, their candidate target genes and the embryogenic potential of the explants was investigated. Our results indicate the involvement of miR165/166-*PHB/PHV* and miR160-*ARF10/ARF16* nodes in regulation of SE. We postulate that miR165/166 and miR160 contribute to the embryogenic transition in Arabidopsis through the indirect impact on the *LEC2* expression and modulation of the auxin biosynthesis in the explant tissue. The results expand our knowledge on the genetic regulation of SE induction and indicate the new components, miR160 and miR165/166, that operate in this auxin-related developmental pathway.

## Results

### Expression level of *PHB* and *PHV* during SE in relation to miR165/166

**Figure 1:**
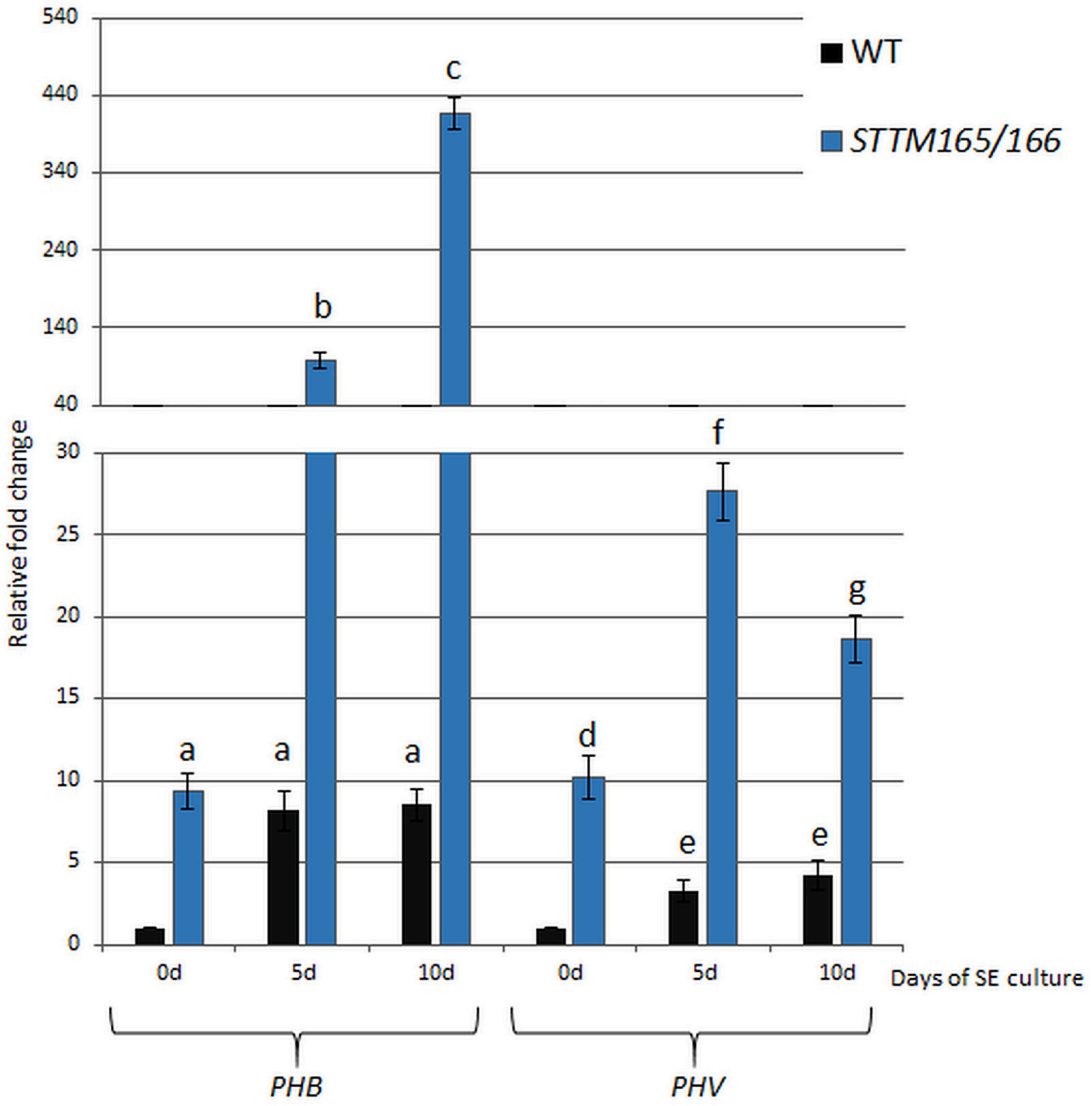
Regulatory relation of miR165/166 and *PHB/PHV* in SE. Expression level of *PHB* and *PHV* in SE culture of WT (Col-0) and *STTM165/166* transgenic line that were induced on medium with 5 μM 2,4-D. Relative transcript level was normalized to the internal control (*At4g27090*) and calibrated to 0 day of the WT culture. Statistical analyses were performed using two-way ANOVA (*P* < 0.05) followed by Tukey's honestly significant difference test (Tukey HSD-test) (*P* < 0.05) in order to assess the differences between the gene expression at 0, 5, and 10 days of the SE culture within a genotype and between genotypes. Significantly different values are indicated by different letters (*P* < 0.05; *n* = 3 ± standard error) SE, somatic embryogenesis; d, day of SE culture.

To test the potential regulatory impact of miR165/166 on somatic embryogenesis (SE), the level of the candidate target transcripts, *PHB* and *PHV*, was evaluated during embryogenic transition induced in Arabidopsis explants. RT-qPCR analyses indicated a significant increase of *PHB* (up to 8-fold) and *PHV* (up to 4-fold) transcripts at the early (5 d) and advanced (10 days) SE stages (Figure 1). We analyzed an SE culture of previously characterized *STTM165/166* line with an abolished miR165/166 function (Yan et al., 2012) and found a strong accumulation (up to 50-fold) of *PHB* and *PHV* transcripts (Figure 1), which suggests a negative impact of miR165/166 on *PHB* and *PHV* expression in SE.

### Expression of *PHB* and miR166 is localized in SE-involved explant parts

**Figure 2:**
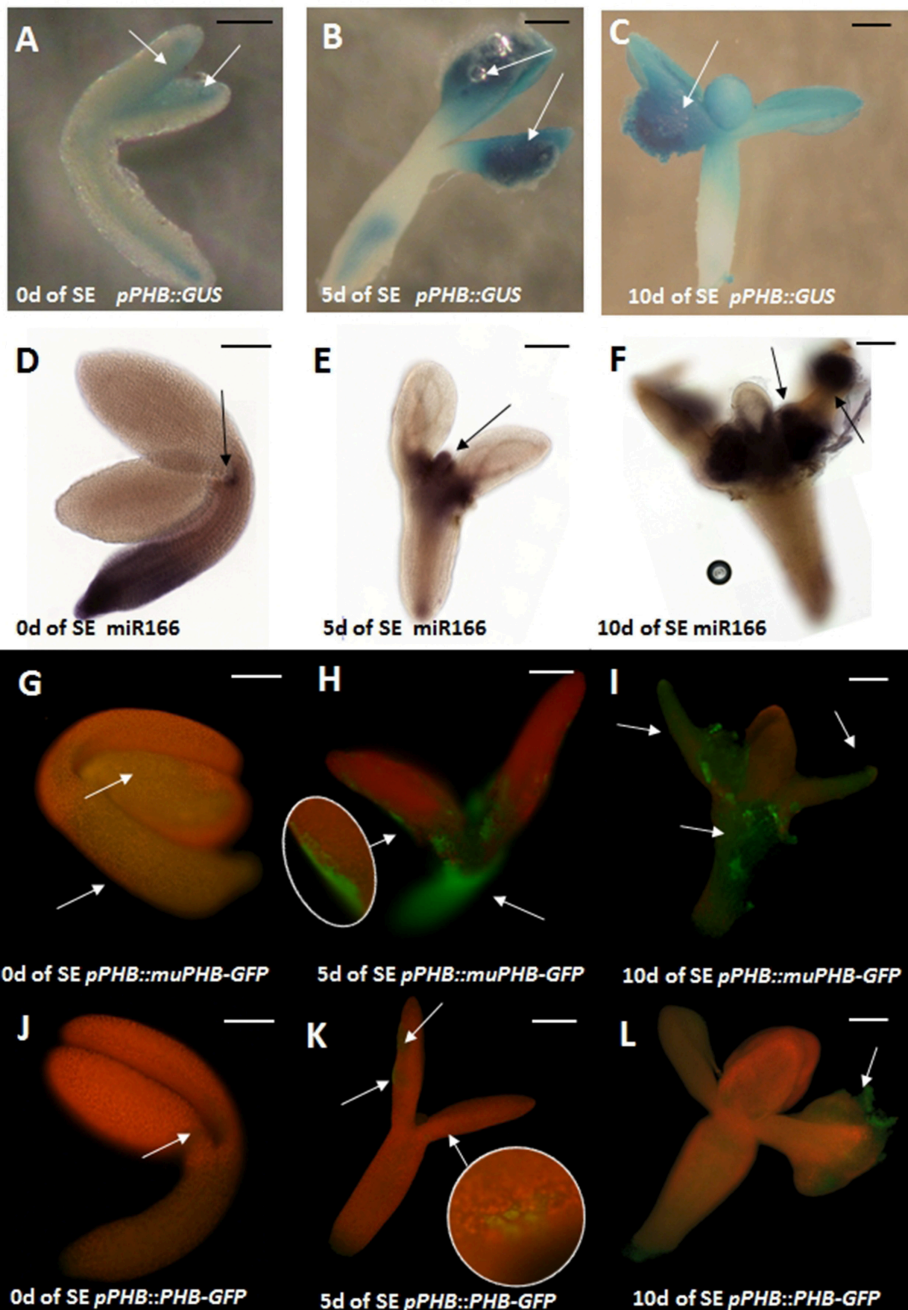
Spatio-temporal expression pattern of miR166 and *PHB* in explants cultured on the SE induction medium with 5 μM 2,4-D at 0, 5, and 10 days (0, 5, 10 d). GUS- **(A–C)** and GFP-**(G–L)** monitored expression of *PHB;* WISH detection of miR166 **(D–F)**. *PHB* expression in the *pPHB::muPHB-GFP* culture carrying a *PHB* transcript that is resistant to miR165/166 **(G-I)** miR165/166-restricted *PHB* expression in the *pPHB::PHB-GFP* culture **(J–L)**. Arrows indicate the GUS/GFP signal associated with the SE-involved tissue. A probe against the mouse miR124 was used as the negative control (Figure S2). Scale bar indicate 100 μm. SE, somatic embryogenesis; d, day of SE culture.

The activity of *PHB* promoter in the embryogenic culture was monitored with the use of the *pPHB::GUS* line. The analysis showed that in freshly isolated explants (0 day) the GUS signal was dispersed across explant and the strongest signal was detected along the adaxial side of the cotyledons (Figure 2A) that is involved in SE induction (Kurczynska et al., 2007). Further intensification of the GUS signal in the cotyledons was observed in the explant undergoing SE induction (5 and 10 days culture) but the *PHB* expression signal was also detected in other explant parts including the root (5 days) and hypocotyl (10 days) that are not involved in SE (Figures 2B,C).

Whole mount RNA *in situ* hybridizations (WISH) with miR166-specific probes were used to examine the spatio-temporal localization of miR166 in explants induced toward SE. The freshly isolated explants (0 day) exhibited strong miR166 signal at the basal explant part including the hypocotyl and the root (Figure 2D). The pattern of miR166 was drastically changed in the explants cultured on SE induction medium and the intensive accumulation of miR166 at the shoot apical meristem (SAM) and its proximity was characteristic of the explants that were cultured for 5 days and at the SAM or cotyledons at the 10th day of SE (Figures 2E,F).

In order to verify the hypothesis that miR165/166 might negatively regulate the *PHB* transcripts during SE induction, we compared the pattern of the GFP signal in *pPHB::PHB-GFP* with the *pPHB::muPHB-GFP* culture expressing *PHB* transcripts that are resistant to the miR165/166 cleavage (Miyashima et al., 2011). Analysis of the GFP signal showed that the *PHB* expression undergoes extensive spatio-temporal changes in the explants of both analyzed lines, but the pattern of PHB signal localization was distinctly different in 5 and 10 days culture of *pPHB::muPHB-GFP* in comparison to *pPHB::PHB-GFP* (Figures 2G–I vs. Figures 2J–L) explants. In the *pPHB::muPHB-GFP* tissue induced toward SE the PHB signal was detected in various explant parts including the hypocotyl, SAM and cotyledons (Figures 2H,I), while in the *pPHB::PHB-GFP* culture, the signal was limited to the cotyledons (Figures 2K,L). The apparent differences in the *PHB* expression pattern in the *pPHB::muPHB-GFP* vs. *pPHB::PHB-GFP* culture include a lack of *PHB* expression in the hypocotyl and root part and less intensive GFP signals in the cotyledons of *pPHB::PHB-GFP* (Figures 2G–I vs. Figures 2J–L).

### A regulatory relationship between miR165/166 and *LEC2* in SE

**Figure 3:**
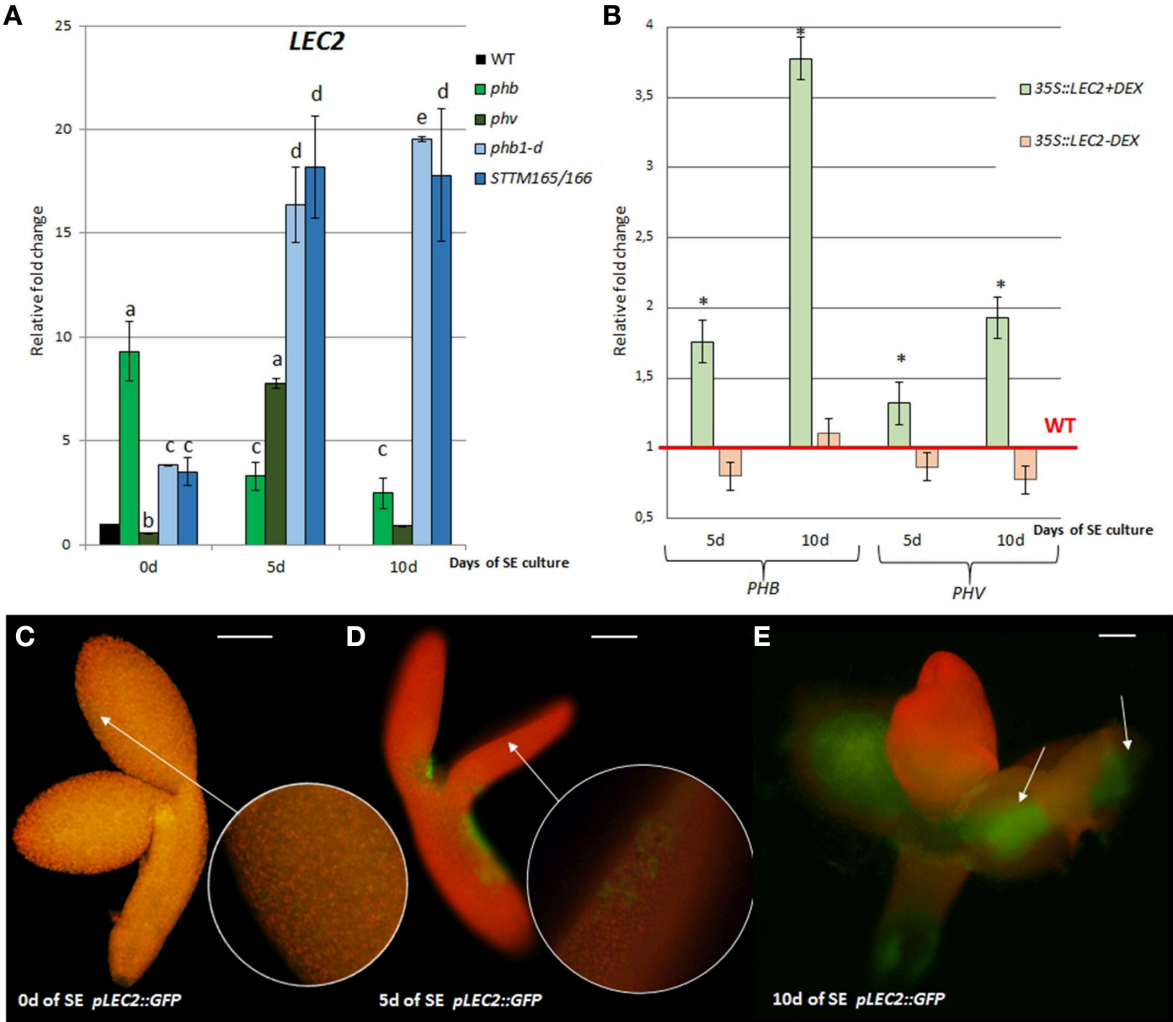
Regulatory relation of miR165/166-*PHB/PHV* with *LEC2* in the SE culture that was induced on the medium with 5 μM 2,4-D. **(A)** Expression level of *LEC2* in the SE culture of *phb, phb1-d, phv*, and *STTM165/166* transgenic line. Relative transcript level was normalized to the internal control (*At4g27090*) and calibrated to 0 day of the WT culture. Statistical analyses were performed using two-way ANOVA (*P* < 0.05) followed by Tukey's honest significant difference test (Tukey HSD-test) (*P* < 0.05) in order to assess the differences between the gene expression at 0, 5, and 10 days of the SE culture within a genotype and between genotypes. Significantly different values are indicated by different letters (*P* < 0.05; *n* = 3 ± standard error) **(B)** Expression level of *PHB* and *PHV* in the SE culture of the DEX inducible *35S::LEC2-GR* line on the medium with (+) or without (–) DEX. Statistical analyses were performed using the *T-*test (*p* < 0.05) to assess the differences between the genotypes. Values that were significantly different from the WT-derived culture of the same age are indicated with an asterisk (*n* = 3 ± standard error). **(C–E)** Localization of *LEC2* expression in SE-induced *pLEC2::GFP* explants that were cultured for 0 **(C)**, 5 **(D)**, and 10 **(E)** days. Scale bar indicates 100 μm. SE, somatic embryogenesis; d, day of SE culture. Arrows indicate *LEC2* expression in the explants areas undergoing SE induction.

In order to investigate a potential relationship between miR165/166-*PHB/PHV* and *LEC2* in SE we analyzed *LEC2* expression levels in embryogenic cultures with disturbed expression of the miR165/166 and *PHB/PHV* transcripts. We found that accumulation of the *PHB* transcripts in the gain-of-function *phb1-d* mutant and a *STTM165/166* line led to the significantly increased *LEC2* transcription (Figure 3A). Further evidence of a regulatory relationship between miR165/166-*PHB/PHV* and *LEC2* was provided by the analysis of the embryogenic culture overexpressing *LEC2* (Figure 3B). We observed that the overexpression of *LEC2* during SE resulted in a significantly increased level of the *PHB* and *PHV* transcripts, thus inferring a positive feedback loop between PHB/PHV and LEC2. In support of this postulate, we found that similar to *PHB*, also *LEC2* is expressed at 5th and 10th day of culture in the cotyledons, i.e., the explant parts that are involved in SE induction (Figures 3C–E). Collectively, these observations support the hypothesis on a regulatory relation between miR165/166-PHB and *LEC2* in the embryogenic transition.

Surprisingly, the up-regulation of *LEC2* transcripts was also noticed in SE culture of the *phb* and *phv* insertional mutants with defected expression of *PHB* (knock-out) and *PHV* (knock-down) genes, respectively (Figure 3A). To explain this result, we found the *PHB* and *PHV* transcripts to be up-regulated in *phv* and *phb* cultures, respectively (Figure S3). In conclusion, the level of the *PHB* and *PHV* transcripts appears to be controlled by a compensative regulatory mechanism in which insufficient expression of one of these genes results in a significantly increased transcription of the other gene.

### A regulatory relationship between miR160 and *ARF10, ARF16*, and *ARF17* in SE

**Figure 4:**
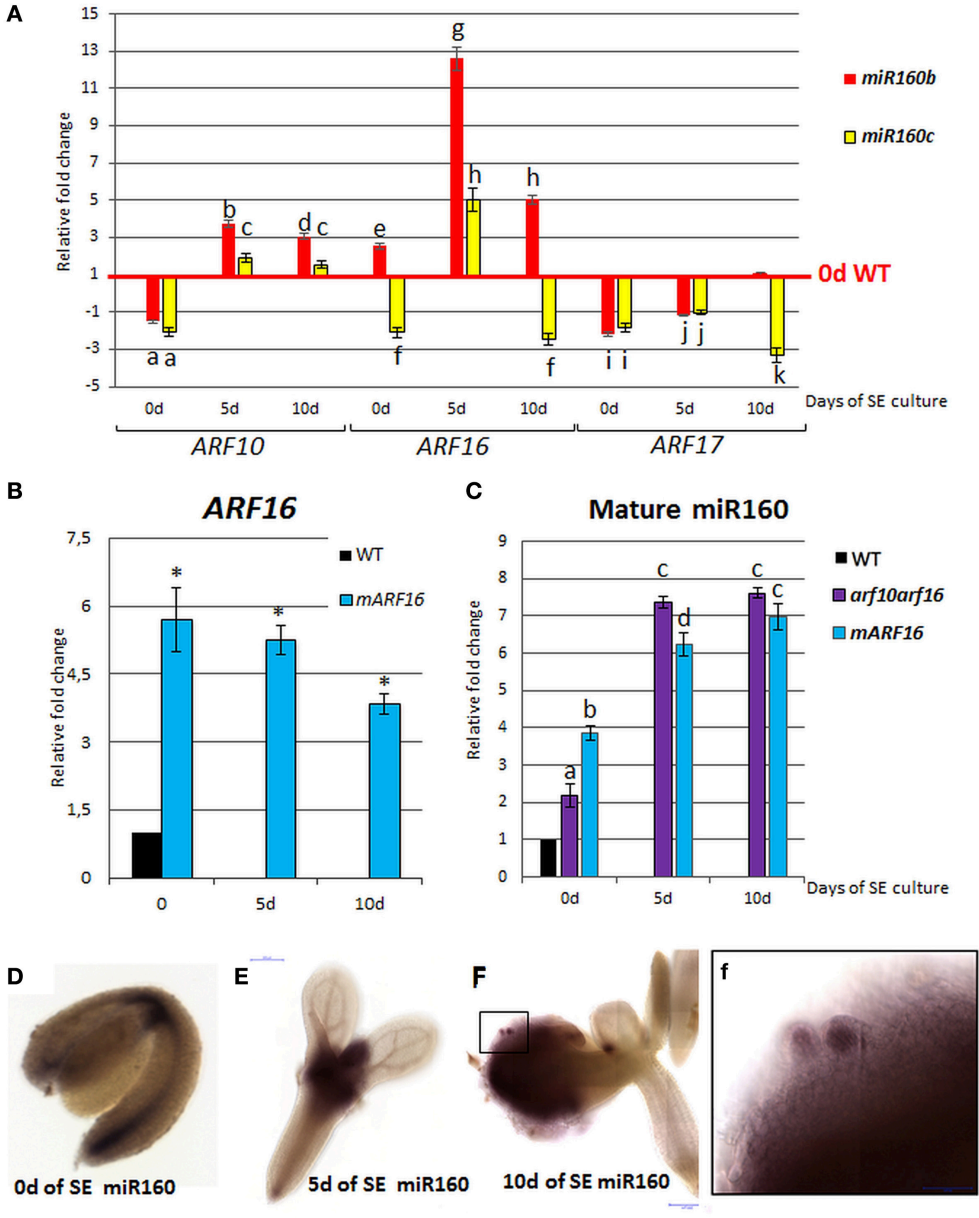
Regulatory relation of miR160 and the target *ARF10, ARF16*, and *ARF17* genes in the SE culture that was induced on the medium with 5 μM 2,4-D. Expression levels of *ARF10, ARF16*, and *ARF17* in the *miR160b* and *miR160c* culture **(A)** *ARF16* in the *mARF16* culture **(B)** miR160 in the *arf10arf16* and *mARF16* cultures **(C)**. WISH detection of miR160 in the explants that were induced toward SE at 0 **(D)**, 5**(E)**, 10 days **(F)**. A higher magnification **(f)** of an area marked in **(F)** showing an accumulation of miR160 in the somatic embryo. Relative transcript level was normalized to the internal control (*At4g27090*) and calibrated to the 0 day of the WT culture. Statistical analyses were performed using two-way ANOVA (*p* < 0.05) followed by Tukey's honest significant difference test (Tukey HSD-test) (*P* < 0.05) in order to assess the differences between gene expression at 0, 5, and 10 days of the SE culture within a genotype and between genotypes. Statistically significant differences (*P* < 0.05) are indicated by different letters (*P* < 0.05; *n* = 3 ± standard error). Statistical analyses (B) were performed using the *T*-test (*P* < 0.05) to assess the differences between the genotypes. Values that were significantly different from the WT-derived 0 day of the culture are indicated with an asterisk (*P* < 0.05; *n* = 3 ± standard error). Probes against the mouse miRNA miR124 were used as the negative controls (Figure S2). SE, somatic embryogenesis; d, day of SE culture.

To verify the assumption of the existence of a regulatory relationship between miR160 and *ARF10/ARF16/ARF17* in SE, the cultures derived from *miR160b* and *miR160c* insertional lines were analyzed. The results indicated that the expression of *ARF10* and *ARF16* was up-regulated in the mutant cultures while the *ARF17* transcription was down-regulated (Figure 4A). In addition, a significantly increased accumulation of the *ARF16* transcripts was observed in cultures from the *mARF16* line that carry the miR160-resistant form of *ARF16* (Figure 4B). A negative feedback loop between *ARF10/ARF16* and miR160 in SE might be suggested as we found an increased miR160 level in the cultures of *mARF16* and *arf10arf16* mutants (Figure 4C).

### WISH localization of miR160 in SE

WISH analysis with a miR160-specific probe indicated that miR160 localization patterns changed in explants during SE. More specifically, the explant at 0 day exhibited miR160 signal along a basal part of the explant while after 5 days of culture on the SE-induction medium, a strong accumulation of miR160 was observed in the SAM and its proximity (Figures 4D–E). At the advanced stage of SE (10 day) the miR160 signal was moved from the SAM area into the embryogenic tissue produced on cotyledons (Figure 4F) and a strong accumulation of miR160 was characteristic of somatic embryos (Figure 4f).

### miR160-*ARF10/16* and *LEC2* interact during SE

**Figure 5:**
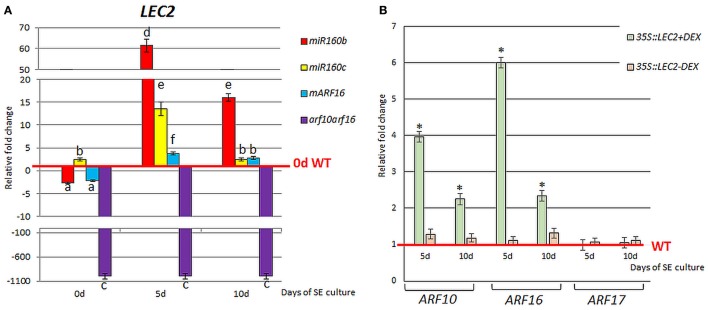
Regulatory relation of miR160-*ARF10/16/17* with *LEC2* in the SE culture that was induced on the medium with 5 μM 2,4-D. Expression level of *LEC2* in the SE culture of *miR160b, miR160c, mARF16*, and *arf10arf16* **(A)**. The relative transcript level was normalized to the internal control (*At4g27090*) and calibrated to 0 day of the WT culture. Statistical analyses were performed using two-way ANOVA (*P* < 0.05) followed by Tukey's honest significant difference test (Tukey HSD-test) (*P* < 0.05) in order to assess the differences between gene expression at 0, 5, and 10 days of the SE culture within a genotype and between genotypes. Statistically significant differences (*P* < 0.05) are indicated by different letters (*P* < 0.05; *n* = 3 ± standard error). **(B)** Expression level of *ARF10, ARF16*, and *ARF17* in the SE culture of the DEX inducible *35S::LEC2-GR* line on the medium with (+) or without (–) DEX. Bars represent standard error. Statistical analyses were performed using the *T*-test (*P* < 0.05) to assess the differences between the genotypes. Values that were significantly different from the WT-derived culture of the same age are indicated with an asterisk (*n* = 3 ± standard error). SE, somatic embryogenesis; d, day of SE culture.

To investigate a relation of miR160 with the LEC2-mediated pathway of SE induction, the expression level of *LEC2* in the embryogenic cultures with impaired expression of miR160 (*miR160b, miR160c*) and its target genes, *ARF10* and *ARF16* (*mARF16, arf10arf16*) was analyzed. *LEC2* transcripts were accumulated in *miR160b, miR160c*, and *mARF16* cultures and down-regulated in *arf10arf16* culture (Figure 5A). Thus, a regulatory relationship between miR160-ARF10/ARF16 and *LEC2* appears to be engaged in SE induction. In addition, a positive impact of *LEC2* on *ARF10/ARF16* cannot be ruled out as we noticed the increased expression of *ARF10* and *ARF16* in the embryogenic culture with *LEC2* overexpression (Figure 5B). In contrast*, ARF17* transcripts was not affected in the *LEC2-* overexpressing culture suggesting that *LEC2* does not regulate *ARF17* during SE.

### miR160 and miR165/166 affect the endogenous auxin content in the SE-induced explants

**Figure 6:**
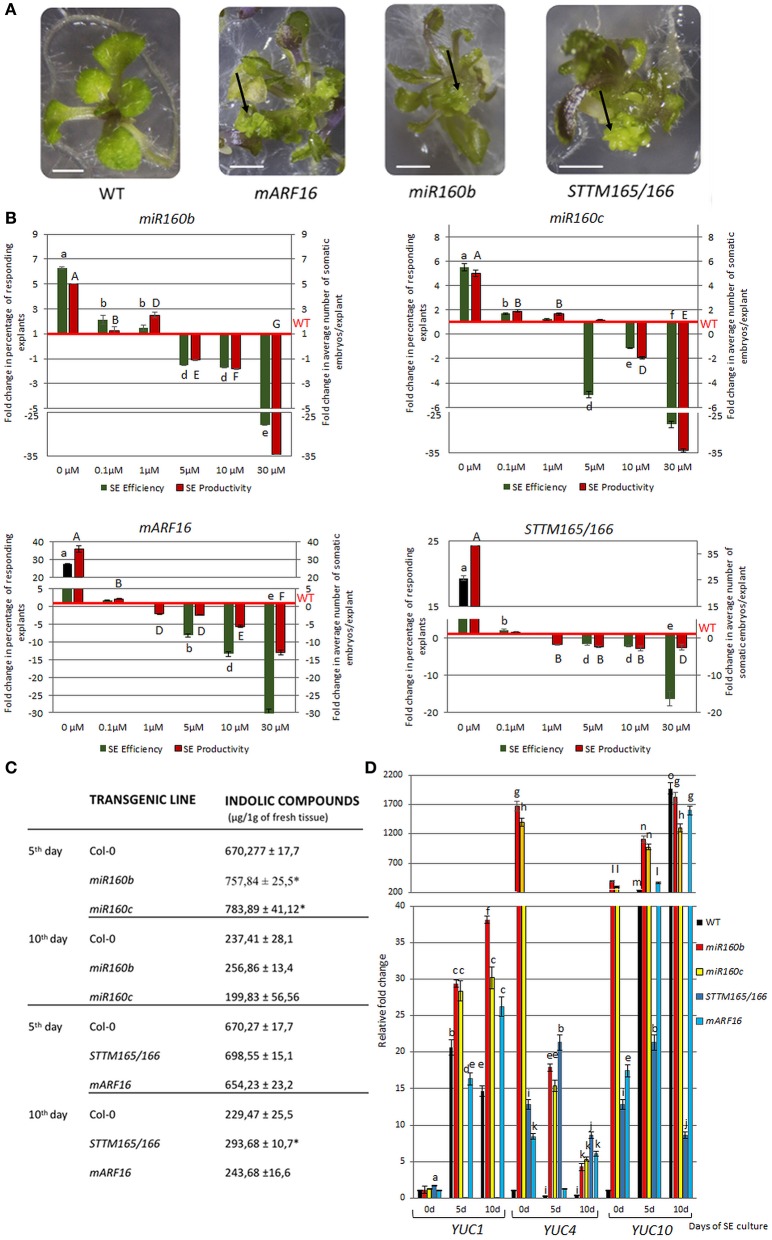
Auxin-related function of miR160 and miR165/166 in the SE culture. The culture of *miR160b, miR160c, mARF16*, and *STTM165/166* explants resulted in somatic embryo formation (marked with arrows) on the auxin-free medium **(A)** 2,4-D concentration-dependent embryogenic potential evaluated as the relative SE efficiency and productivity **(B)**. SE efficiency and productivity was calibrated to WT culture. Statistical analyses were performed using two-way ANOVA (*P* < 0.05) followed by Tukey's honest significant difference test (Tukey HSD-test) (*P* < 0.05) in order to assess the differences between the effect of different 2,4-D concentrations within a genotype and between genotypes. Statistically significant differences (*P* < 0.05) are indicated by different lower case letters (SE efficiency) or uppercase letters (SE productivity). Equivalent means have the same letter (*P* < 0.05; *n* = 3 ± standard error). **(C)** The content of indolic compounds in the *miR160b, miR160c, mARF16*, and *STTM165/166* explants during SE. Statistical analyses were performed using the *T*-test (*P* < 0.05) to assess the differences between the genotypes. Values that were significantly different from the WT-derived culture are indicated with asterisks (*P* < 0.05; *n* = 3 ± standard error). **(D)** Expression of *YUC* (*YUC1, YUC4*, and *YUC10*) genes that are involved in auxin biosynthesis in the *miR160b, miR160c, mARF16*, and *STTM165/166* explants during SE. The relative transcript level was normalized to the internal control (*At4g27090*) and calibrated to the 0 day of the WT culture. Statistical analyses were performed using two-way ANOVA (*P* < 0.05) followed by Tukey's honest significant difference test (Tukey HSD-test) (*P* < 0.05) in order to assess the differences between gene expression at 0, 5, and 10 days of the SE culture and between genotypes. Statistically significant differences (*P* < 0.05) are indicated by different letters (*P* < 0.05; *n* = 3 ± standard error). Scale bar indicates 1 cm. The SE culture **(C,D)** was induced on the medium with 5 μM 2,4-D.

The regulatory relationships of miR160 and miR165/166 with the *LEC2* gene, a key regulator of SE through the control of auxin biosynthesis (Wójcikowska et al., 2013), suggested that the biological function of these miRNAs during SE might be related with auxin response. Consistent with this hypothesis, we observed that the embryogenic response that was displayed by the mutants with a disrupted expression and function of miR160 and miR165/166 on the auxin media was significantly different to the WT culture. That is, the *miR160b, miR160c, mARF16*, and *STTM165/166* explants produced somatic embryos on the auxin-free medium and supplementation of the medium with 2,4-D drastically impaired their embryogenic response in a concentration-dependent manner (Figures 6A,B; Table S1). Thus, we hypothesized that miR160 and miR165/166 impact the auxin content in the SE-induced explants. In support of this hypothesis, we found that the levels of IAA-related indolic compounds (Bric et al., 1991) were significantly higher in the cultures of *miR160b, miR160c*, and *STTM165/166* (Figure 6C). In addition, we found that the representative auxin-inducible AUX/IAA genes, *IAA17*, and *IAA29* (Overvoorde et al., 2005), were up-regulated in the SE cultures with a reduced expression of miR160 (*miR160b, miR160c*) or an increased expression of the presumptive targets (*STTM165/166, mARF16*) (Figure S4). Because our results indicated that miR160 and miR165/166 control the *LEC2* that has a regulatory role in the *YUCCA* (*YUC*)-mediated pathway of auxin biosynthesis during SE (Wójcikowska et al., 2013), we profiled the expression of *YUC* genes in *STTM165/166, mARF16, miR160b*, and *miR160c* cultures. We found that *YUC1, YUC4*, and *YUC10* genes were highly up-regulated in these cultures (Figure 6D). Collectively, our results suggest that miR160 and miR165/166 may contribute to the embryogenic potential of Arabidopsis somatic tissues via the regulation of the LEC2-controlled pathway of auxin biosynthesis.

### Regulatory relationship of miR160 and miR166/165 pathways during SE

**Figure 7:**
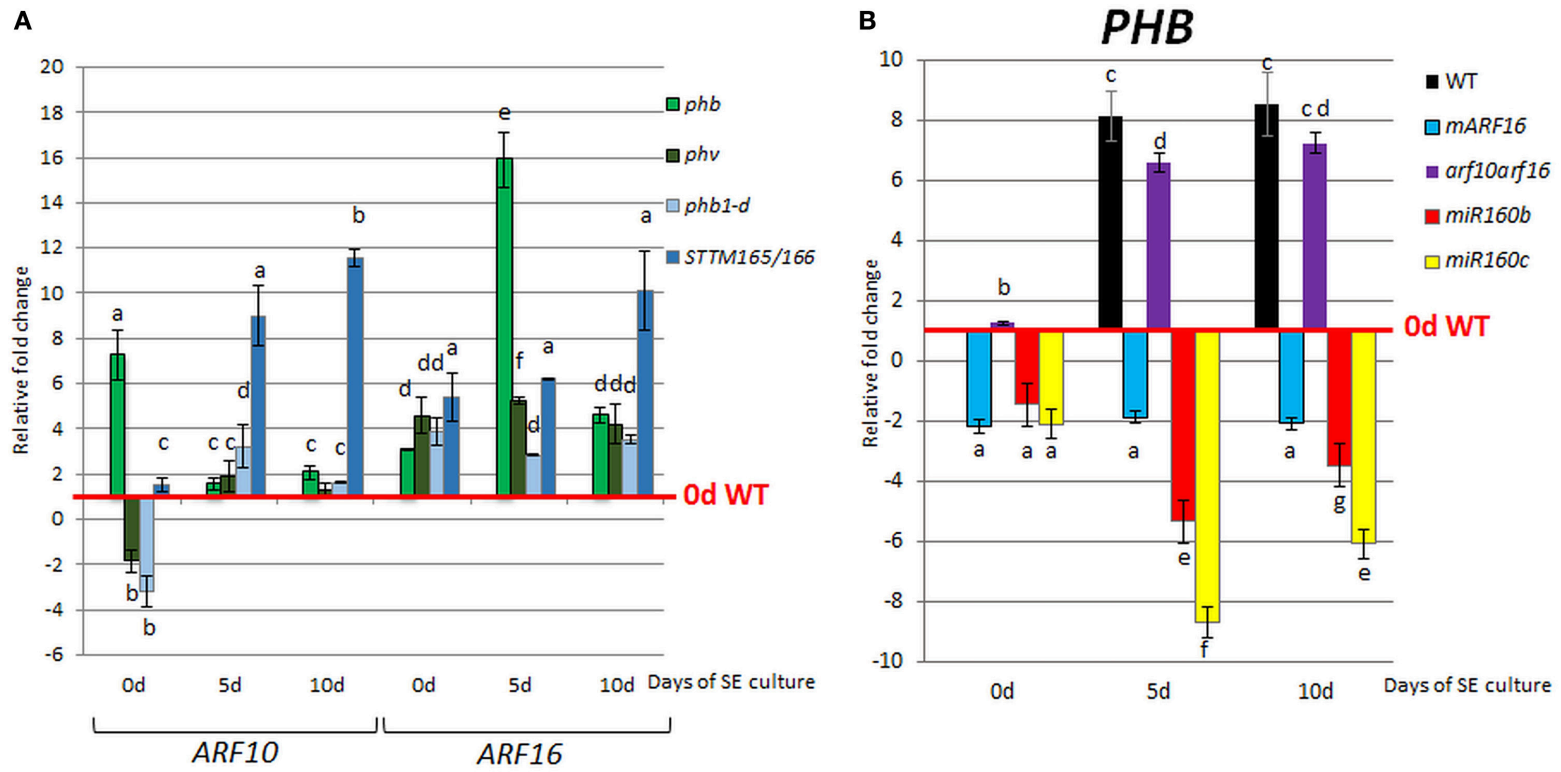
Regulatory relationship of miR165/166 and miR160 nodules. Expression level of *ARF10* and *ARF16* in *phb, phv, phb1-d*, and *STTM165/166* **(A)** and *PHB* in *mARF16, arf10arf16, miR160b*, and *miR160c* **(B)** SE culture that was induced on the medium with 5 μM 2,4-D. The relative transcript level was normalized to the internal control (*At4g27090*) and calibrated to the 0 day of the WT culture. Statistical analyses were performed using two-way ANOVA (*P* < 0.05) followed by Tukey's honest significant difference test (Tukey HSD-test) (*P* < 0.05) in order to assess the differences between gene expression at 0, 5, and 10 days of the SE culture within a genotype and between genotypes. Statistically significant differences (*P* < 0.05) are indicated by different letters (*P* < 0.05; *n* = 3 ± standard error).

Given that both miR160 and miR165/166 impact *LEC2* expression during SE we then investigated the regulatory relationships between presumptive miR160 (*ARF10, ARF16*) and miR165/166 (*PHB/PHV*) targets. We found that the level of *ARF10* and *ARF16* transcripts was significantly increased in the *phb, phv, phb1-d* and *STTM165/166* lines (Figure 7A), thus suggesting that *PHB* might positively affect *ARF10* and *ARF16* expression. Moreover, *PHB* was up-regulated in the culture of *arf10arf16* double mutant, and down-regulated in *mARF16, miR160b* and *miR160c* indicating that *ARF10* and *ARF16* might negatively control *PHB* expression (Figure 7B).

**Figure 8:**
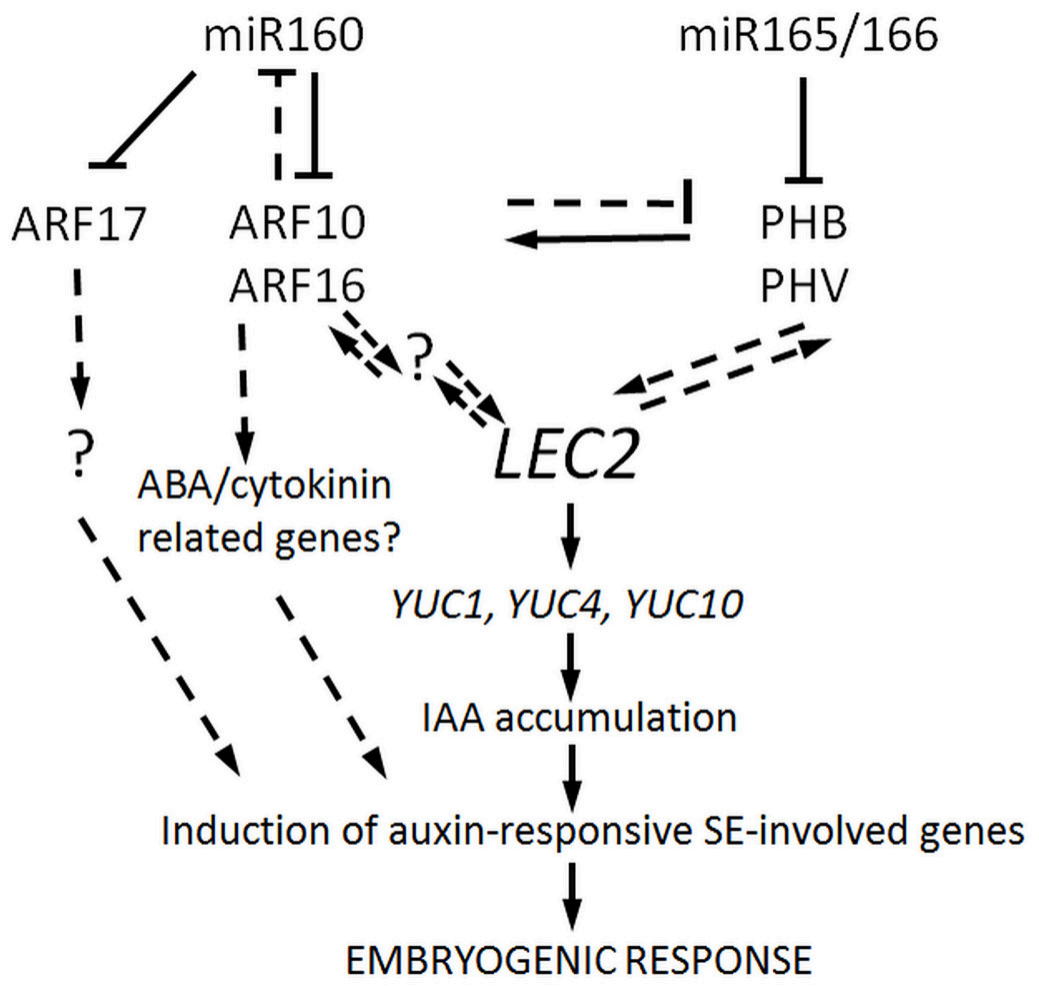
A model of miR160- and miR165/166-regulated pathways contributing to SE induction. *ARF10/ARF16* and *PHB/PHV* that are repressed by miR160 and miR165/166, respectively, are postulated to positively regulate LEC2, thus controlling SE induction via the up-regulation of *YUC* genes (*YUC1,4*, and *10*) and the activation of the auxin biosynthesis pathway. As a consequence of IAA accumulation, the auxin-responsive genes that are involved in SE induction are triggered. The targets of miR160 and miR165/166 appear to interact via a negative feedback loop as PHB/PHV seems to positively control their repressors, ARF10/ARF16. Besides controlling the auxin response, ARF10/ARF16 might also contribute to SE induction by impacting the signaling pathways of other hormones including ABA and/or cytokines. The role of ARF17 in SE induction appears to be unrelated to the LEC2-mediated pathway. Further analyses are required to validate the assumed intra- and inter-specific regulatory interactions, which are indicated with dashed lines and to identify any other genetic components that are involved and are marked with a question mark.

Altogether our results suggest that miR160 and miR165/166, possibly through the regulation of *ARF10/ARF16* and *PHB/PHV*, respectively, contribute to the SE induction mechanism associated with LEC2-controlled auxin biosynthesis pathway (Figure 8). However, further experiments are necessary to determine the mode of interaction between the miR160 and miR165/166 pathways during SE induction and to further elaborate the gene regulatory networks that they are involved in.

## Materials and methods

### Plant material

The *A. thaliana* (L.) Heynh. Columbia (Col-0) and *Landsberg erecta* (Ler) parental genotypes and the transgenic lines *miR160b, miR160c, phb, phv, phb1-d* and *pPHB::GUS* were supplied by Nottingham Arabidopsis Stock Centre (NASC). The T-DNA insertional double mutants *arf10-2 arf16-2* (hereafter noted as *arf10arf16*) and *P*_*ARF*16_*::mARF16* (hereafter noted *mARF16*) were kindly provided by Prof. Xiaoya Chen (Institute of Plant Physiology and Ecology, China). The seeds of *2x35S::STTM165/166* line (hereafter noted as *STTM165/166*) were kindly provided by Dr. Guiliang Tang (Michigan Technological University, USA). The *pPHB::PHB-GFP* and *pPHB::muPHB-GFP* line that carries the mutated, resistant to the miR165/166 cleavage version of the *PHB* transcript (Miyashima et al., 2011), were kindly provided by Prof. Keiji Nakajima (Nara Institute of Science and Technology, Japan). The *35S::LEC2-GR* line overexpressing *LEC2* upon DEX treatment was described previously (Ledwon and Gaj, 2009). To monitor expression of *LEC2* the *pLEC2::GFP* line with nuclear localized GFP was generated by cloning the *LEC2* promoter sequence (−2020 to +5 relative to ATG of *LEC2*) into pCR™8/GW/TOPO® (Invitrogen), and then recombining these plasmids with Gateway-compatible pCGTAG plasmids using LR clonase (Invitrogen). Col-0 plants were transformed with use of the floral dip method (Clough and Bent, 1998).

The characteristics of the transgenic lines that were used in the study are presented in Table S2A.

### Plant growth conditions

Seeds sterilized with 20% solution of commercial bleach were plated on 0.5x MS solid medium. The plates were kept at 4°C in darkness for 2 days and then transferred to a growth chamber at 21 ± 1°C under a 16/8h photoperiod of 40 μM m^−2^s^−1^ white, fluorescent light. The young seedlings were transplanted into Jiffy-7 pots and grown in a walk-in type green room under the conditions described above until harvesting of siliques.

### Somatic embryogenesis induction

Immature zygotic embryos (IZEs) at green cotyledonary-stage were used as explants to induce SE. IZEs were cultured in E5 solid medium with 5 μM 2,4-dichlorophenoxyacetic acid (2,4-D) according to Gaj (2001). In some experiments (Figures 4A,B), other concentrations of 2,4-D were used including 0; 0.1; 1.0; 10.0; 30.0 μM. The explant capacity for SE was evaluated in 21 day-culture and two parameters were calculated: SE efficiency (the percentage of explants that formed somatic embryos) and SE productivity (the average number of somatic embryos per explant). All of the culture combinations were evaluated in three replicates and at least 30 explants (ten explants/Petri dish) were analyzed per one replicate.

### Whole mount *in situ* hybridization of miRNAs

The whole mount *in situ* hybridization (WISH) of miRNA molecules was performed according to modified protocol of Dastidar et al. (2016). Embryos/explants were dissected/collected in a drop of PBS (Phosphate-buffered saline buffer) and immediately transferred to 4% paraformaldehyde on ice. LNA Digoxin 5'-end labeled probes were used following to the Dastidar et al. (2016). The slides were scanned using Panoramic FLASH 250 II.

### RNA isolation and RT-qPCR analysis

An RNAqueous kit (Ambion) was used to isolate total RNA and mirVana^TM^ Kit was used to isolate miRNAs from the IZE explants. Depending on the age of a culture, 300 (0 day culture) to 100 (10 days culture) explants were collected for RNA/miRNA isolation. The concentration and purity of RNA was evaluated with a ND-1000 spectrophotometer (Nano-Drop). To avoid DNA contamination, RNA was treated with RQ1 RNase-free DNase I (Promega) following the manufacturer's instructions. First strand cDNA was produced using a RevertAid First Strand cDNA Synthesis Kit (Fermentas). RT-qPCR was carried out in a 10 L^−1^ reaction volume using a LightCycler 480 SYBR Green I Master (Roche). The primers that were relevant to the genes being studied were used in the RT-qPCR analysis (Table S2B). The RT-qPCR reactions were performed as previously described in Wójcik and Gaj (2016). Primary data analysis was performed using LightCycler Software (Roche).

### Detection of mature miRNA

The oligonucleotides design, stem-loop RT and real time qPCR were performed according to Speth and Laubinger (2014). The primers sequences used in the study are listed in Table S2B. Primary data analysis was performed using LightCycler Software (Roche). Relative RNA levels were calculated and normalized to an internal control of the *At4g27090* gene encoded 60S ribosomal protein (Thellin and Zorzi, 1999). In all of the analyzed tissue samples, the control gene displayed a constant expression pattern with Cp = 19 ± 1. The plant tissues for the analysis of gene expression were produced in three biological replicates and two technical replicates of each repetition were carried out. The relative expression level was calculated using 2^−ΔΔ*Ct*^.

### Histological analysis

To detect GUS signal the *pPHB::GUS* explants were stained with a standard X-Gluc (Sigma Aldrich) solution at 37°C for 12 h according to Jefferson et al. (1987). The tissue was inspected under Delta-Optical SZ-630T microscope and images were saved as jpg files with a Canon EOS 60D camera.

Analysis of GFP signal was carried out using a Nikon Eclipse Ni-E/Ni-U fluorescent microscope system. GFP fluorescence was excited with halogen lamphouses with a 100–240 VAC (Prior Lumen200) and a wavelength of 488 nm. Photographic documentation was recorded by Nikon Digital Sight DS-Fi2 with DS-U3 camera, using the NIS-Elements F computer program version 4.0.

### Evaluation of indolic compounds level

A colourimetric technique that enabled the detection of indolic compounds, including IAA, was applied (Bric et al., 1991; Wójcikowska et al., 2013). To evaluate the relationship between IAA content and miR160 and miR166 activity, IZE-derived cultures of Col-0 and *miR160b, mARF16, STTM165/166* genotypes were analyzed. IZE explants were induced on a 5μM 2,4-D medium and tissues were sampled on the 5th or 10th day of culture. The procedure was performed as described previously (Wójcikowska et al., 2013). Each analysis was carried out in three biological replicates.

### Statistical analysis

The statistical analyses were performed using either the Student *t*-test or a two-way ANOVA (*p* < 0.05) followed by Tukey's honestly significant difference test (Tukey HSD-test) (*p* < 0.05). The figures show the averages from at least three biological replicates with the standard error.

## Discussion

Experimental and technological advances have recently provided evidence that in animals and plants dynamically fine-tuned expression of *TRANSCRIPTION FACTORS* (*TFs*) accounts for the correct pattern formation in the embryo developing from the zygotic cell (Jeong et al., 2012; Bedzhov et al., 2014). In embryogenic cultures of Arabidopsis the extensive changes in the transcriptomes of SE-induced cells that involve the profound modulation of both the *TF* transcripts and *MIRNA*/miRNA were indicated (Le et al., 2010; Xiang et al., 2011; Lara-Chavez et al., 2012; Gliwicka et al., 2013; Seefried et al., 2014; Szyrajew et al., 2017). Thus, it is expected that in SE, similarly to zygotic embryogenesis (ZE) (Nodine and Bartel, 2010), the miRNA-directed transcriptional regulation of the embryonic TFs might play the essential function. Consistent with this postulate, we studied the functions of miR160 and miR165/166 in embryogenic culture of Arabidopsis.

### miR165/166 contributes to the SE induction via *PHB/PHV* regulation

During ZE, miR165/166-mediated repression of the *PHB* and *PHV* genes enables the morphogenesis-to-maturation transition and correct specification of shoot apical meristem (SAM) and root apical meristem (RAM) in the developing embryo (Grigg et al., 2009; Miyashima et al., 2013). Relevant to zygotic development, our results suggest that miR165/166 restricts *PHB/PHV* expression to control embryonic development in Arabidopsis somatic cells cultured *in vitro*. In support of this postulate, we found that the expression profile of *PHB/PHV* is inverse to the one that was indicated for miR165/166 in the WT embryogenic culture (Szyrajew et al., 2017) (Figure S1A). In line with this finding, we showed the *PHB* and *PHV* transcripts to be accumulated in the culture of the *STTM165/166* lines with an abolished miR165/166 function. In order to further test the regulatory relationship between miR165/166 and *PHB*, the spatio-temporal patterns of *PHB* and miR166 were investigated in SE-induced explants with use of reporter lines (*pPHB::GUS; pPHB::PHB-GFP*) and WISH, respectively. Our results indicated changes in the localization of PHB signal and miR166 during SE induction. Although PHB and miR166 both accumulate in the proximity of the shoot apical meristem (SAM), the area that is involved in SE induction (Kurczynska et al., 2007), their expression patterns appear to not overlap. Accordingly, cotyledons that are predominantly involved in somatic embryo formation displayed a strong expression of *PHB* while the WISH signal of miR166 was limited to the SAM and its proximity. Further evidence that miR166 might spatio-temporally repress the *PHB* transcripts in SE-induced tissue was supported by the observation of a more widespread and intense *PHB* expression in the *pPHB::muPHB-GFP* than in the *pPHB::PHB-GFP* culture (Figures 2G–I vs. Figures 2J–L). miR165/166 appears to spatiotemporally restrict the *PHB* transcripts in the explant parts/cells that are not responsive to SE induction. In contrast, the SE-responsive cells that are dispersed along the explant cotyledons (Kurczynska et al., 2007) seem to accumulate *PHB* transcripts (Figures 1, 2K,L), which in part, might result from the decreased content of miR166 (Szyrajew et al., 2017). However, more advanced cytohistological analysis is required to evaluate the relation between the miR165/166 content and the *PHB* transcript level in the explant cells that are undergoing SE induction.

Moreover, consistent with the finding that the *pPHB::PHBmu-GFP* plants phenocopy the miR165/166-resistant *phb1-d* mutant (Miyashima et al., 2013), we found that the *pPHB:muPHB-GFP* line showed a reduced SE response, which was similar to the *phb1-d* mutant (Figure S5). The impaired SE response of *pPHB:muPHB-GFP* and *phb1-d* explants on an auxin medium that is standard for SE-induction is possibly caused by an increased IAA accumulation, which might result from the upregulation of *LEC2* (Figure 3A) and the activation of auxin biosynthesis *YUC* genes (Figure 6D). A relation between the endogenous auxin accumulation and the impaired embryogenic potential of tissue on a standard auxin medium was indicated for different genotypes in the present (Figure 6B) and other studies (Wójcikowska et al., 2013; Wójcik and Gaj, 2016). Altogether, the analysis of the reporter lines suggests that in SE-induced explants miR166 might restrict *PHB* expression to the cotyledon tissue and the resulting spatial distribution of *PHB* expression colocalizes with explant sites developing somatic embryos.

The rapid and intense accumulation of *PHB* transcripts in SE-involved explant parts that we observed in response to SE induction medium suggests that PHB might be involved in the very early events associated with the embryogenic transition of somatic cells. The genetic regulation of early events associated with embryogenic transition in the *in vitro* cultured somatic cells are poorly known and identification of the genes acting up- and down-stream from *PHB* in the very early SE culture might provide new insights into molecular mechanism of SE induction.

### miR160 impacts SE via control of auxin signaling regulators, *ARF10/ARF16/ARF17*

Recently, the impact of miR160 on developmental processes induced *in vitro* was reported. Accordingly, miR160-mediated repression of *ARF10/ARF16/ARF17* was postulated to control the embryogenic response in culture of *D. longan* (Lin et al., 2015) and miR160-ARF10 was shown to control cellular reprogramming and callus formation in Arabidopsis (Liu et al., 2016). Our results indicate that miR160 might also contribute to SE induction in Arabidopsis through the repression of *ARF10, ARF16*, and *ARF17*, in light of (i) the opposite expression patterns of *ARF10/ARF16/ARF17* and miR160 in the Arabidopsis embryogenic culture (Szyrajew et al., 2017; Wójcikowska and Gaj, 2017) (Figure S1B); (ii) the increased expression of *ARF10* and *ARF16* in the *miR160* mutant cultures and the accumulation of *ARF16* in the cultures expressing the miRNA-resistant version of *ARF16*. Differences in the accumulation of *ARF10/ARF16* at distinct time points of the *miR160* mutants SE culture may be caused by a redundancy between the members of the *MIR160* family, all of which are able to cleave transcripts of *ARF10* and *ARF16* (http://plantgrn.noble.org/psRNATarget/).

The unimpaired SE response in *arf10* and *arf16* single mutants compared to the impaired SE response of double *arf10arf16* mutants (Figure S5) suggests that ARF10 and ARF16 seem to function redundantly during SE. In support of this postulate, the redundant function of ARF10 and ARF16 was indicated in root cap formation (Wang et al., 2005) and seed dormancy (Liu et al., 2013) and recently, ARF10/ARF16 together with IAA17 were found to act as a protein complex (Ye et al., 2016).

Interestingly, we observed the significant accumulation of miR160 in *mARF16* and *arf10arf16* cultures that suggests a role of *ARF16* in the control of miR160 during SE. The feedback regulation of miRNA production by the associated target genes was reported for mammalian and plant miRNAs (Wu et al., 2009; Lai et al., 2016). Importantly for the present result, miR160 was found to be negatively regulated by the targeted *ARF17* to control auxin homeostasis and adventitious rooting in Arabidopsis (Gutierrez et al., 2009). Thus, it cannot be excluded that during SE in Arabidopsis miR160 is controlled by the targeted *ARF*s but this assumption needs further experimental verification.

The distinct differences in the level of the primary transcripts of *MIR160a, MIR160b*, and *MIR160c* in Arabidopsis embryogenic culture (Szyrajew et al., 2017) suggest that *MIR160* genes contribute differently to the regulation of SE. In line with this assumption we found *ARF10* and *ARF16* but not *ARF17* to be accumulated in *miR160b* and *miR160c* mutant cultures. This result implies that during SE *ARF17* might be under the control of *MIR160a*. In support for this postulate, exclusively *MIR160a* was indicated to control *ARF17* during early development of zygotic embryo (Liu et al., 2010). The analysis of *ARF17* expression in the SE culture with defected *MIR160a* expression would verify this hypothesis but the severely reduced fertility of the *foc* mutant (*miR160a*) (Liu et al., 2010) makes isolation of immature zygotic embryos and thus the establishment of SE culture difficult.

The assumption that a role of *ARF17* in SE might be different to *ARF10/ARF16* supports also the finding that in embryogenic culture expression of *ARF17*, in contrast to *ARF10/ARF16*, is not affected by *LEC2* overexpression. Moreover, the expression pattern of *ARF17* in SE differs from *ARF10/ARF16* and accordingly, a level of *ARF17* mRNAs increases in late SE and is auxin-independent (Wójcikowska and Gaj, 2017). Thus, it cannot be ruled out that miR160-*ARF17* operates in the advanced SE culture associated with the formation of somatic embryos. Suggestive for this postulate is formation of the defective zygotic embryos in plants with suppressed miR160-directed regulation of *ARF17* (Mallory et al., 2005).

### miR160 and miR165/166 control SE induction via LEC2-stimulated pathway of auxin biosynthesis

Importantly for a role of miR160- and miR165/166-regulated pathways in SE, we found that transgenic forms with defective expression and function of miR160 (*miR160b, miR160c, mARF16*) and miR165/166 (*STTM165/166*) were capable to produce somatic embryos on auxin-free medium and auxin treatment severely impaired their embryogenic response. Similar capacity for SE induction on auxin-free medium displayed also the culture overexpressing *LEC2* and accumulated IAA (Ledwon and Gaj, 2011; Wójcikowska et al., 2013). Similar to the culture overexpressing *LEC2*, in embryogenic culture of the *miR160* and *STTM165/166* we found increased accumulation of the indolic compounds and enhanced expression of *LEC2* that was coupled with activation of the *YUC* (*YUC1, YUC4*, and *YUC10*) genes encoding the auxin biosynthesis enzymes involved in SE induction in Arabidopsis (Wójcikowska et al., 2013).

The results imply that miR165/166 might control SE induction by impacting the *PHB* and *PHV* genes that encode the direct activators of *LEC2* (Tang et al., 2012). Although the PHB and PHV are closely related and demonstrate the high degree of functional interchangeability (McConnell et al., 2001; Prigge et al., 2005) the mechanism adjusting their individual contribution to the controlled processes, including SE, has not been yet revealed.

In support for the LEC2-related function of PHB in SE we observed the up-regulated expression of *LEC2* in embryogenic culture of *phb-1d* mutant that was reported to spontaneously produce somatic embryos (Tang et al., 2012). Moreover, we found *LEC2* overexpression to enhance *PHB/PHV* transcription level suggesting that a positive feedback regulation exists between *PHB/PHV* and *LEC2* during SE. In support of this assumption, we have identified the RY- motif recognized by LEC2 (Braybrook et al., 2006) in the *PHB* promoter (AGRIS Atcis DB) but the experimental verification for the direct binding of LEC2 to the *PHB* promoter during SE would be necessary to test this further. Interestingly, the LEC2-binding RY-motif is also present in the promoters of the *MIR165/166* genes (Wang and Perry, 2013) and thus it is conceivable that *LEC2* might also control expression of *MIR165/166* genes during SE. So far, few TFs directly regulating *MIRNA* genes have been implicated in plants including activation of *MIR165a* and *MIR166b* by SHR (SHORT ROOT) and SCR (SCARECROW) during post-embryonic development (Carlsbecker et al., 2010; Miyashima et al., 2013). LEC2 regulation of *MIRNA* genes has not been reported yet but it cannot be excluded as FUS3, a TF structurally and functionally related with LEC2 (Harada, 2001), was suggested to control *MIR156, MIR160, MIR166, MIR396* genes in the embryogenic culture of Arabidopsis (Wang and Perry, 2013).

Altogether, several lines of evidence infer that the miR165/166-*PHB/PHV* regulatory node controls induction of the embryogenic program in somatic cells of Arabidopsis through targeting *LEC2*. The possible role of miR165/166 in the regulation of HD-ZIP III TFs during SE was also postulated in sweet orange and *Larix leptolepis* but the targeted effectors and molecular pathways controlled were not identified (Wu et al., 2011; Li et al., 2013).

Our results suggest that miR165/166-*PHB/PHV* and miR160-*ARF10*/*ARF16* regulatory modules might regulate SE induction through LEC2. Accordingly, the significant changes in *LEC2* expression levels in SE cultures with a disturbed expression and function of the *ARF10* and *ARF16* genes (*mARF16* and *arf10arf16*) suggest that these ARFs positively regulate *LEC2*. In addition, ARF10 and ARF16 seem to contribute to *LEC2* regulation in SE due to the auxin-stimulated expression of *LEC2* and the similarity of spatio-temporal expression pattern of *LEC2* and *ARF10/ARF16* in embryogenic culture (Kurczynska et al., 2007; Ledwon and Gaj, 2009; Wójcikowska and Gaj, 2017). Considering that ARF10 and ARF16 repress auxin-regulated genes (Guilfoyle and Hagen, 2007) they are unlikely to control *LEC2* directly and the intermediary genetic elements remain to be identified.

In conclusion, both the miR165/166- *PHB/PHV* and miR160- *ARF10/ARF16* nodes might control the embryogenic transition in Arabidopsis somatic cells via regulating *LEC2*, which is a key regulator of SE induction. The convergent functions of miR160 and miR165/166 in regulation of a common TF, the *WOX5* (*WUSCHEL-RELATED HOMEOBOX 5*) gene, was reported in control of distal stem cell differentiation and embryonic root development (Grigg et al., 2009; Ding and Friml, 2010). Given that a role of *WOX5* in formation of RAM in somatic embryos of Arabidopsis was reported (Su et al., 2015; Wang and Chong, 2016) the contribution of miR160 and miR165/166 to SE through regulation of the embryonic root development might be considered.

In addition to controlling the auxin biosynthesis-related *LEC2* gene, ARF10/ARF16 might also impact SE induction via the regulation of the genes that are involved in the signaling of other hormones including ABA. Accordingly, miR160-ARF10 was found to play an important role in ABA-auxin crosstalk in seed germination and post-embryonic developmental programs (Liu et al., 2007). ARF16 was indicated to be required for the *ABI3* expression (Liu et al., 2013) that encodes a transcription factor that is involved in ABA signaling during seed development (Finkelstein et al., 2002). It is worth noting that an *abi3-1* mutant that was insensitive to ABA was found to be significantly impaired in its SE response (Gaj et al., 2006). These findings together with the extensive interactions between auxin and ABA signaling during plant development (Rock and Sun, 2005; Teale et al., 2008; Thole et al., 2013) including the induction of SE in Arabidopsis (Braybrook and Harada, 2008) infer that the disturbed ABA sensitivity that is expected in *mARF16* might enhance auxin perception/signaling and as a result, an embryogenic response is triggered. Recently, the role of the ARF10-miR160 module in the regulation of cytokinin-auxin crosstalk was indicated (Liu et al., 2016). Important for the SE-induction mechanism, overexpression of miR160 was shown to enhance tissue sensitivity to cytokines (Turner et al., 2013), which were reported to play a key role in auxin-induced SE in carrot (Tokuji and Kuriyama, 2003) and Arabidopsis (Su et al., 2015; Wang and Chong, 2016).

The regulatory interaction between miR165/166 and miR160 in SE seems to include the negative feedback loop between *ARF10/ARF16* and *PHB/PHV*, the targets of miR160 and miR165/166 pathways, respectively. Experimental supports for this notion include: (i) a positive impact of *PHB* on the *ARF10/ARF16* expression level (increased *ARF10/ARF16* transcription in *phb, phv, phb1-d, STTM165/166* (ii) a negative relation of ARF10/ARF16 on *PHB* transcription in the *mARF16, miR160b* and *miR160c* cultures. The higher expression of *ARF10* and *ARF16* in the *STTM165/166* than in the *phb1-d* culture may be caused by an increased level of miR160, which is able to cleave *ARF10/ARF16* transcripts (Wang et al., 2005) in *phb1-d* (Figure S6A). In support of a possible role of PHB in the direct activation of *ARF10, ARF16* in SE, a binding of PHB to *ARF5* promoter during vascular patterning in Arabidopsis was documented (Müller et al., 2015). However, it is also possible that ARF16 might repress *PHB* via up-regulation of miR166 as we found a decreased level of miR166 in *arf10arf16* culture (Figure S6B).

## Conclusions

Our results indicate that miR160 and miR165/166-regulated pathways distinctly contribute to the regulation of developmental plasticity of Arabidopsis cells under *in vitro* conditions. Accordingly, miR160 and miR165/166 through targeting *ARF10/ARF16* and *PHB/PHV*, respectively, were found to impact the SE induction through the LEC2-mediated auxin-biosynthesis pathway. In this scenario, the repression of both miR160 and miR165/166 leads to a higher expression of *LEC2*, which results in the *YUC*-mediated biosynthesis of auxin. As a consequence, IAA accumulates in explant tissues that trigger auxin responsive genes involved in the embryogenic transition.

Beside impacting the auxin biosynthesis miR165/166 might also contribute to the embryogenic transition via regulation of stress-related genes due to involvement of miR165/166 in modulation of abiotic stress responses (Jia et al., 2015) and a pivotal function of stress responses in SE induction mechanism (Zavattieri et al., 2010; Jin et al., 2014; Fehér, 2015). Thus, the SE-regulators might be also searched among the stress-related genes targeted by miR165/166.

This study provides a significant step forward in understanding the miRNA-mediated mechanism regulating developmental plasticity of plant somatic cells (Garcia, 2008; Rubio-Somoza and Weigel, 2011). The validation of the postulated regulatory interactions that act within and between the miR160- and miR165/166-regulated pathways and identification of other directly and indirectly controlled targets is essential to fully define the miRNA-mediated genetic network controlling SE induction.

## Author contributions

MG and AW conceived and designed the research. AW conducted the experiments; MN provided the WISH of miRNA. MG and AW analyzed the data and wrote the manuscript. All the authors read and approved the manuscript.

### Conflict of interest statement

The authors declare that the research was conducted in the absence of any commercial or financial relationships that could be construed as a potential conflict of interest.

## Supplementary material

Figure S1 The opposite expression patterns of miR165/166 vs. *PHB, PHV* **(A)** and miR160 vs. *ARF10, ARF16, ARF17* **(B)** in the SE culture of WT (Col-0) according to the results for the expression level of miR160, miR165/166 (Szyrajew et al., 2017) and *ARF10, ARF16, ARF17* (Wójcikowska and Gaj, 2017). Relative transcript level was normalized to the internal control (*At4g27090*) and calibrated to the 0 day of culture (*n* = 3); SE, somatic embryogenesis; d, day of SE culture.

Figure S2 A negative control of WISH analysis of miRNA. A probe against mouse miR124 was used in analyses of WT (Col-0) explants cultured on SE-induction medium for 0 **(A)** 5 **(B)** and 10 **(C)** days; SE, somatic embryogenesis; d, day of SE culture.

Figure S3 Expression profile of *PHB* and *PHV* in the SE culture of the *phb* and *phv* mutants that were induced on the medium with 5 μM 2,4-D. The relative transcript level was normalized to the internal control (*At4g27090*) and calibrated to the WT culture. Statistical analyses were performed using two-way ANOVA (*P* < 0.05) followed by Tukey's honest significant difference test (Tukey HSD-test) (*P* < 0.05) in order to assess the differences between gene expression at 0, 5, and 10 days of the SE culture within a genotype and between genotypes. Statistically significant differences (*P* < 0.05) are indicated by different letters (*P* < 0.05; *n* = 3 ± standard error). SE, somatic embryogenesis; d, day of SE culture.

Figure S4 Expression level of the auxin-inducible *IAA17* and *IAA29* genes in the SE culture of the WT, *miR160b, miR160c, mARF16*, and *STTM165/166* lines that were induced on the medium with 5 μM 2,4-D. The relative transcript level was normalized to the internal control (*At4g27090*) and calibrated to 0 days of the WT culture. Statistical analyses were performed using two-way ANOVA (*P* < 0.05) followed by Tukey's honest significant difference test (Tukey HSD-test) (*P* < 0.05) in order to assess the differences between gene expression at 0, 5, and 10 d of the SE culture within a genotype and between genotypes. Statistically significant differences (*P* < 0.05) are indicated by different letters (*P* < 0.05; *n* = 3 ± standard error). SE, somatic embryogenesis; d, day of SE culture.

Figure S5 Functional test of the *arf10arf16, pPHB::muPHB-GFP*, and *phb1-d* lines. The embryogenic potential of the *arf10arf16, pPHB::muPHB-GFP* **(A)** and *phb1-d* **(B)** lines and parental (WT) genotypes on the medium with 5 μM 2,4-D was measured by SE efficiency and SE productivity. Statistical analyses were performed using the *T*-test (*P* < 0.05) to assess the differences between the genotypes. Values that were significantly different from the WT culture are indicated with asterisks (*n* = 3 ± standard error).

Figure S6 Expression level of miR160 **(A)** and miR166 **(B)** in the SE culture of the *phb1-d, STTM165/166*, and *arf10arf16* transgenic lines that were induced on the medium with 5 μM 2,4-D. The relative transcript level was normalized to the internal control (*At4g27090*) and calibrated to the WT culture. **(A)** Statistical analyses were performed using two-way ANOVA (*P* < 0.05) followed by Tukey's honest significant difference test (Tukey HSD-test) (*P* < 0.05) in order to assess the differences between the level of miR160 at 0, 5, and 10 days of the SE culture within a genotype and between genotypes. Statistically significant differences (*P* < 0.05) are indicated by different letters (*P* < 0.05; *n* = 3 ± standard error). **(B)** Statistical analyses were performed using the *T*-test (*P* < 0.05) to assess the differences between the genotypes. Values that were significantly different from the WT-derived culture are indicated with an asterisk (*n* = 3 ± standard error).
