# Supplementary figures and images for: miR160 and miR166/165 Contribute to the LEC2-Mediated Auxin Response Involved in the Somatic Embryogenesis Induction in Arabidopsis
Source: Front Plant Sci. 2017 Dec 11;8:2024. doi: 10.3389/fpls.2017.02024 (PMC5732185; doi:10.3389/fpls.2017.02024)

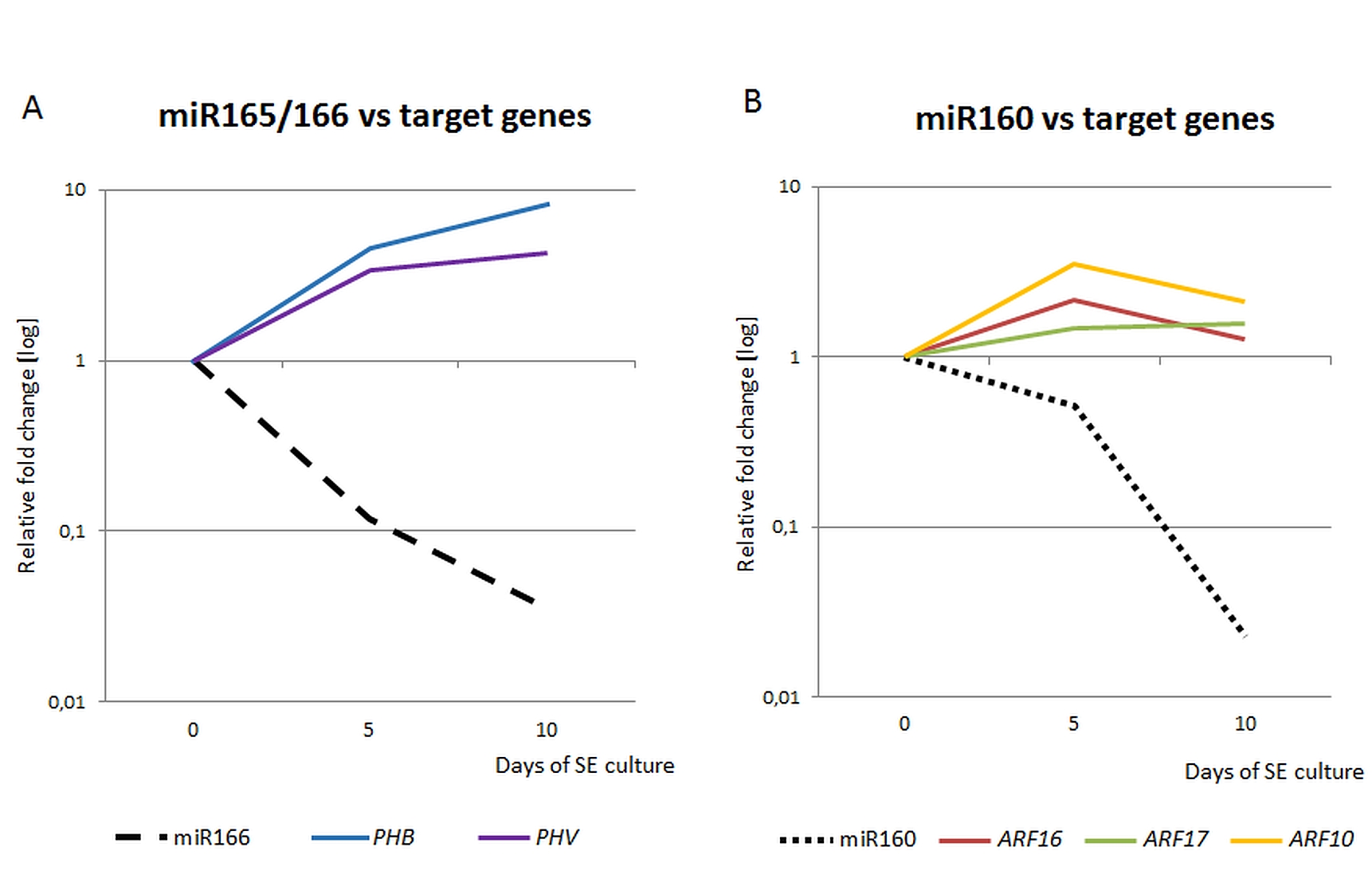

Supplement: Figure S1 — The opposite expression patterns of miR165/166 vs. PHB, PHV (A) and miR160 vs. ARF10, ARF16, ARF17 (B) in the SE culture of WT (Col-0) according to the results for the expression level of miR160, miR165/166 (Szyrajew et al., 2017) and ARF10, ARF16, ARF17 (Wójcikowska and Gaj, 2017). Relative transcript level was normalized to the internal control (At4g27090) and calibrated to the 0 day of culture (n = 3); SE, somatic embryogenesis; d, day of SE culture. [file Image1.JPEG]

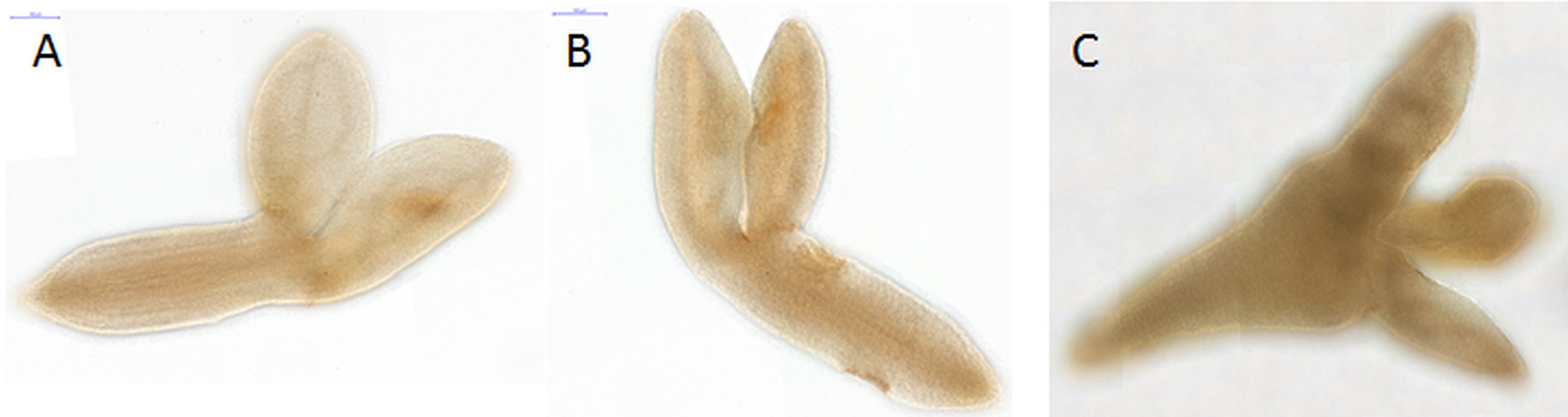

Supplement: Figure S2 — A negative control of WISH analysis of miRNA. A probe against mouse miR124 was used in analyses of WT (Col-0) explants cultured on SE-induction medium for 0 (A) 5 (B) and 10 (C) days; SE, somatic embryogenesis; d, day of SE culture. [file Image2.JPEG]

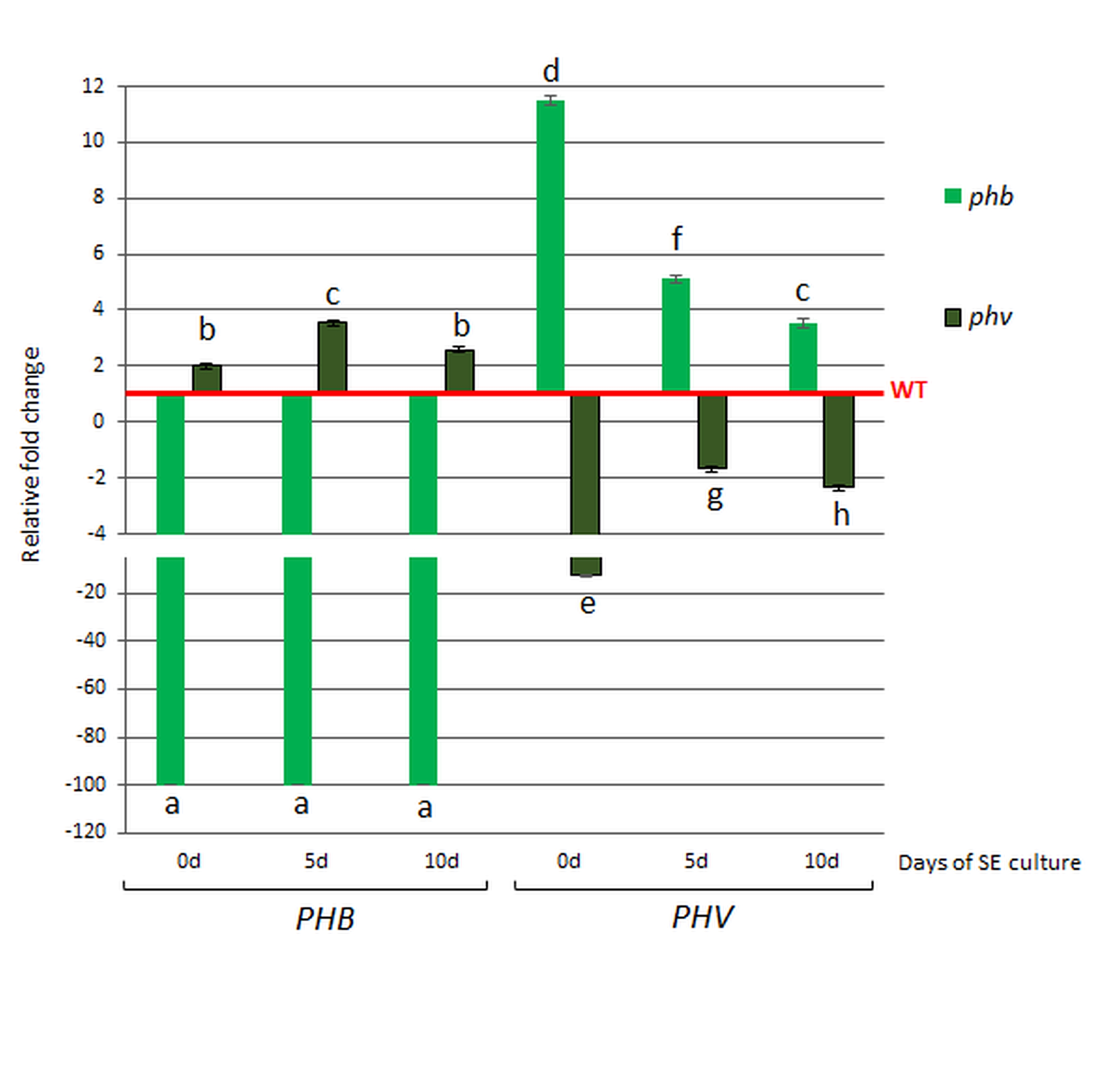

Supplement: Figure S3 — Expression profile of PHB and PHV in the SE culture of the phb and phv mutants that were induced on the medium with 5 μM 2,4-D. The relative transcript level was normalized to the internal control (At4g27090) and calibrated to the WT culture. Statistical analyses were performed using two-way ANOVA (P < 0.05) followed by Tukey's honest significant difference test (Tukey HSD-test) (P < 0.05) in order to assess the differences between gene expression at 0, 5, and 10 days of the SE culture within a genotype and between genotypes. Statistically significant differences (P < 0.05) are indicated by different letters (P < 0.05; n = 3 ± standard error). SE, somatic embryogenesis; d, day of SE culture. [file Image3.JPEG]

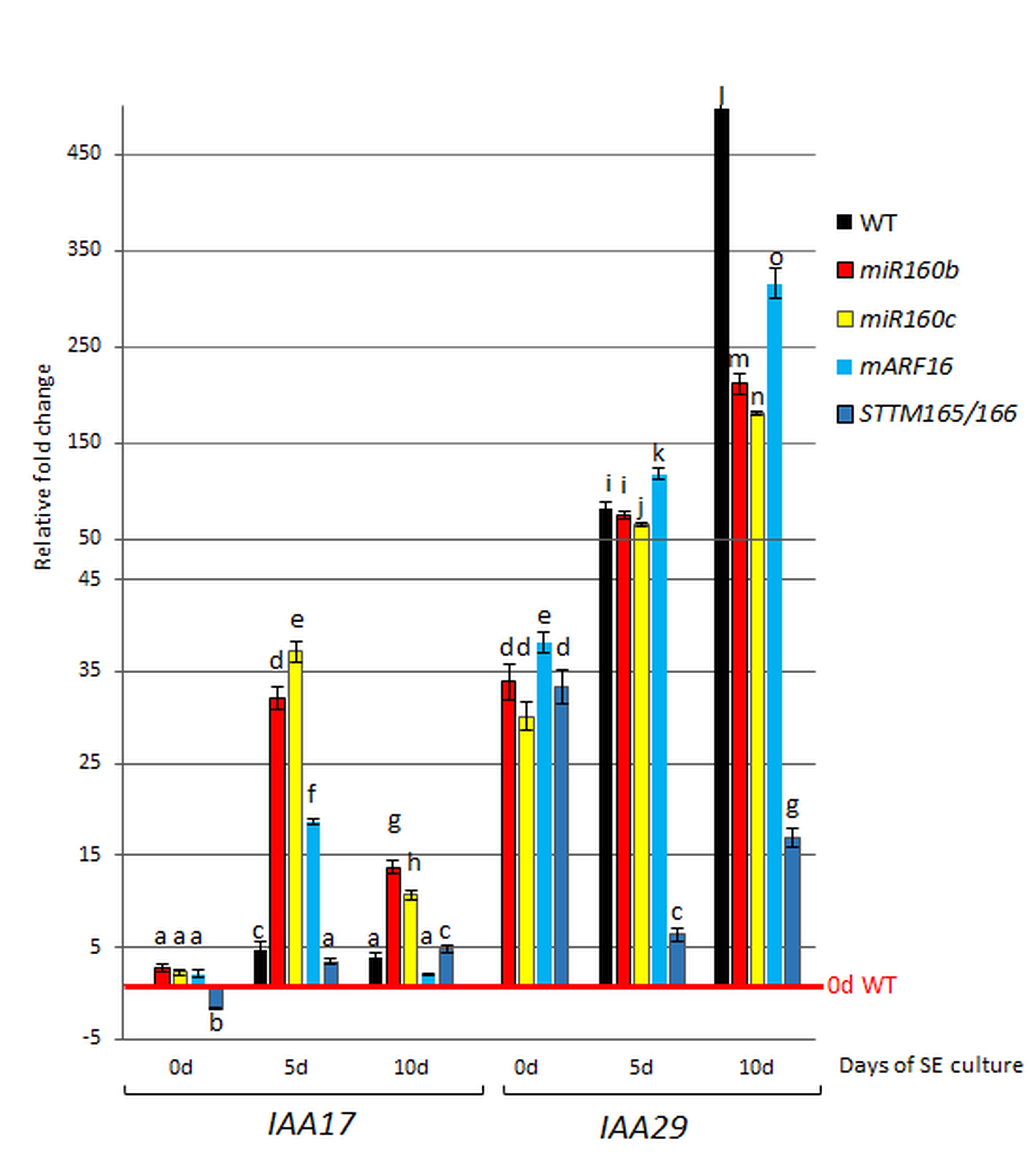

Supplement: Figure S4 — Expression level of the auxin-inducible IAA17 and IAA29 genes in the SE culture of the WT, miR160b, miR160c, mARF16, and STTM165/166 lines that were induced on the medium with 5 μM 2,4-D. The relative transcript level was normalized to the internal control (At4g27090) and calibrated to 0 days of the WT culture. Statistical analyses were performed using two-way ANOVA (P < 0.05) followed by Tukey's honest significant difference test (Tukey HSD-test) (P < 0.05) in order to assess the differences between gene expression at 0, 5, and 10 d of the SE culture within a genotype and between genotypes. Statistically significant differences (P < 0.05) are indicated by different letters (P < 0.05; n = 3 ± standard error). SE, somatic embryogenesis; d, day of SE culture. [file Image4.JPEG]

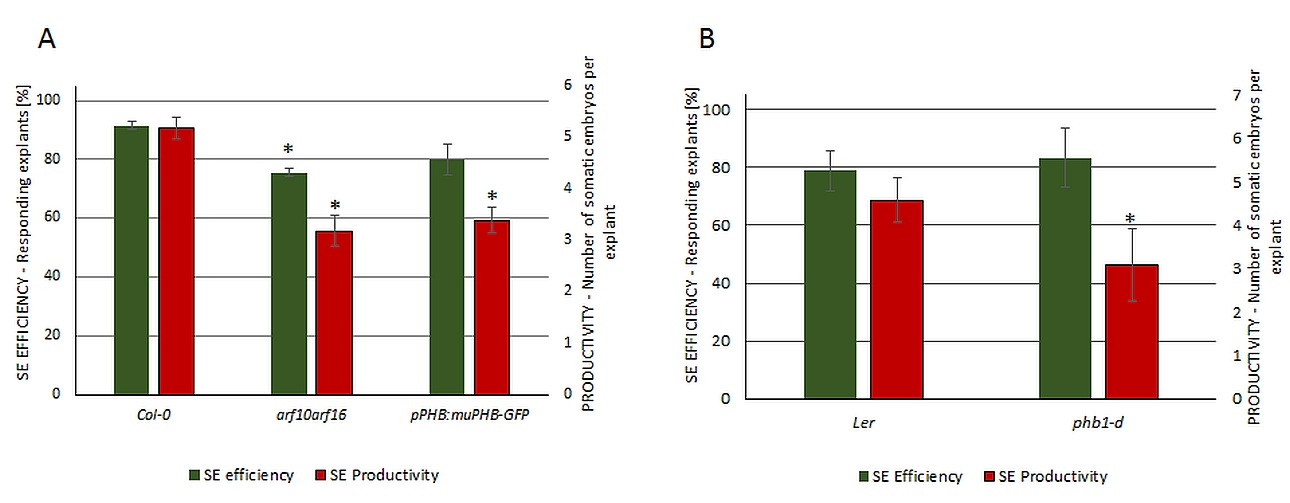

Supplement: Figure S5 — Functional test of the arf10arf16, pPHB::muPHB-GFP, and phb1-d lines. The embryogenic potential of the arf10arf16, pPHB::muPHB-GFP (A) and phb1-d (B) lines and parental (WT) genotypes on the medium with 5 μM 2,4-D was measured by SE efficiency and SE productivity. Statistical analyses were performed using the T-test (P < 0.05) to assess the differences between the genotypes. Values that were significantly different from the WT culture are indicated with asterisks (n = 3 ± standard error). [file Image5.JPEG]

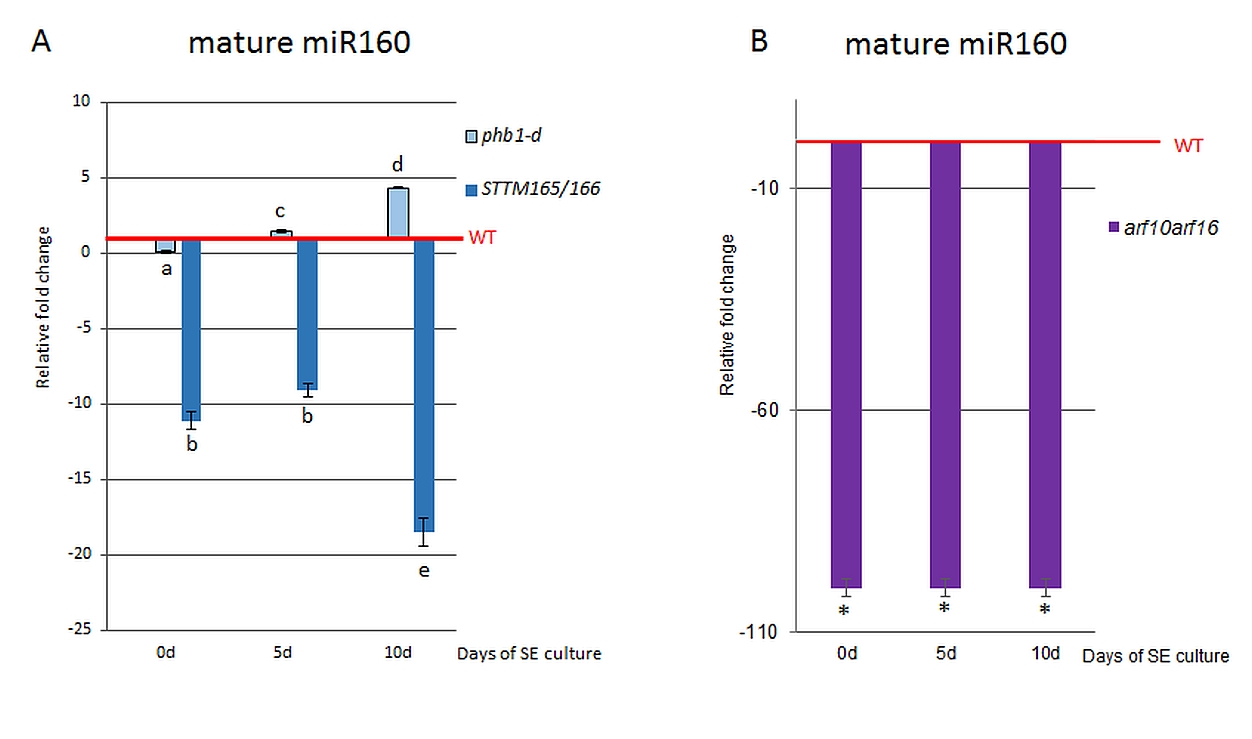

Supplement: Figure S6 — Expression level of miR160 (A) and miR166 (B) in the SE culture of the phb1-d, STTM165/166, and arf10arf16 transgenic lines that were induced on the medium with 5 μM 2,4-D. The relative transcript level was normalized to the internal control (At4g27090) and calibrated to the WT culture. (A) Statistical analyses were performed using two-way ANOVA (P < 0.05) followed by Tukey's honest significant difference test (Tukey HSD-test) (P < 0.05) in order to assess the differences between the level of miR160 at 0, 5, and 10 days of the SE culture within a genotype and between genotypes. Statistically significant differences (P < 0.05) are indicated by different letters (P < 0.05; n = 3 ± standard error). (B) Statistical analyses were performed using the T-test (P < 0.05) to assess the differences between the genotypes. Values that were significantly different from the WT-derived culture are indicated with an asterisk (n = 3 ± standard error). [file Image6.JPEG]
